# Developing Barbed Microtip-Based Electrode Arrays for Biopotential Measurement

**DOI:** 10.3390/s140712370

**Published:** 2014-07-10

**Authors:** Li-Sheng Hsu, Shu-Wei Tung, Che-Hsi Kuo, Yao-Joe Yang

**Affiliations:** Department of Mechanical Engineering, National Taiwan University, Taipei 10617, Taiwan; E-Mails: lshsux@mems.me.ntu.edu.tw (L.-S.H.); tungshuwei@mems.me.ntu.edu.tw (S.-W.T.); chk@mems.me.ntu.edu.tw (C.-H.K.)

**Keywords:** barbed microtips, contact impedance, detaching force, dry electrode, electrocardiography (ECG), electroencephalogram (EEG), silicon wet etching

## Abstract

This study involved fabricating barbed microtip-based electrode arrays by using silicon wet etching. KOH anisotropic wet etching was employed to form a standard pyramidal microtip array and HF/HNO_3_ isotropic etching was used to fabricate barbs on these microtips. To improve the electrical conductance between the tip array on the front side of the wafer and the electrical contact on the back side, a through-silicon via was created during the wet etching process. The experimental results show that the forces required to detach the barbed microtip arrays from human skin, a polydimethylsiloxane (PDMS) polymer, and a polyvinylchloride (PVC) film were larger compared with those required to detach microtip arrays that lacked barbs. The impedances of the skin-electrode interface were measured and the performance levels of the proposed dry electrode were characterized. Electrode prototypes that employed the proposed tip arrays were implemented. Electroencephalogram (EEG) and electrocardiography (ECG) recordings using these electrode prototypes were also demonstrated.

## 1. Introduction

Human organ activity, including that in the brain, eyes, muscles, and heart, generates electrical signals. Biopotential measurement techniques, such as electroencephalogram (EEG), electrocardiogram (ECG), and electromyography (EMG), are vital medical and research tools that analyze human conditions or activities by measuring these electrical signals [1–5]. These signals are typically detected using electrodes attached to specific locations on human bodies [6,7]. Electrolytic gel is typically employed to improve the electrical conductivity of the interface between the electrodes and human skin. Thus, electrodes that require electrolytic gel are often referred to as wet electrodes. Skin abrasion is often applied to the outer skin layer before attaching electrodes to improve the skin-electrode conductivity. These skin preparation procedures for biopotential measurement can become uncomfortable or time consuming [8].

To avoid the inconveniences caused by conventional wet electrodes, various studies have proposed dry electrodes that require no electrolytic gel or skin preparation [9–14]. Microtip arrays, which are fabricated using micromachining techniques, have attracted substantial attention [15], as have biopotential recording methods that involve applying microtips to human skin. Ng *et al.* [16] demonstrated multiple micro-spike electrodes for use in EEG measurement. Each electrode consisted of a micro-pillar, including a microtip on top. Dias *et al.* [17] proposed a dry electrode that comprised 16 microtip structures to apply stimulation and measure biopotential. In [18], the development of micromolded 3D microelectrode arrays for use in transcutaneous nerve-tracking applications was presented. Matteucci *et al.* [19] demonstrated the fabrication of high aspect ratio micropatterned electrodes built with a combination of deep X-ray lithography (DXRL), electroforming, and soft lithography.

Silicon microtips can be fabricated using various etching techniques, such as anisotropic wet etching and dry isotropic/anisotropic etching methods. The etching rates of dry etching methods have little dependence on the orientation of silicon crystal planes [20]. Different types of tip structures have been successfully realized by dry etching methods with appropriate masks and process designs [21–23]. However, compared with wet etching processes, dry etching techniques are typically expensive. Wet silicon anisotropic etching is generally performed in an alkaline etchant. Because the etching rate strongly depends on the crystal orientation, microtip structures can be generated that are defined by the slow etching planes.

To generate tip arrays that effectively adhere to skin, various studies have proposed methods of creating barbs on tips. Griss *et al.* [24] developed vertical barbed spikes by using a series of deep reactive ion etching (RIE) steps. The influence of the shape of barb types on the detachment forces for non-biological materials was also discussed. Byun *et al.* [25] generated barbed in-plane micro-spikes by using deep silicon etching in micro-scale biopsy applications. The barbs on these proposed microtip arrays indeed increased the required detachment forces. However, the fabrication processes for such barbed microtips are typically either complex or expensive.

In this work, a simple fabrication process using low-cost wet etching techniques was proposed for generating electrodes that comprise barbed microtip-based arrays. KOH anisotropic silicon wet etching was employed to generate standard pyramidal microtip arrays. Subsequently, HF/HNO_3_ isotropic etching was used to form the microtip barbs. Also, a method for creating a through-silicon via (TSV), which improves the conductivity between the electrode lead and the tip array, is also proposed. In addition, the required forces of the barbed microtip arrays for detaching from different materials will be measured and discussed. The impedances of the skin-electrode interface will also be studied. The demonstrations of EEG and ECG recordings using electrode prototypes assembled with these arrays will be presented.

## 2. Design and Principle

**Figure 1.**
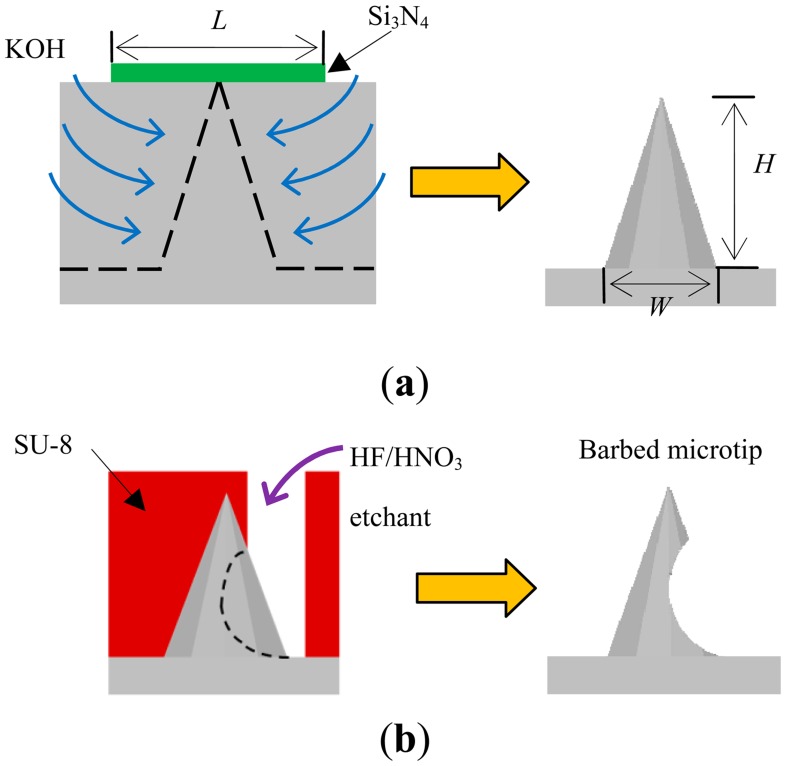
Schematics of a microtip after (**a**) anisotropic and (**b**) isotropic wet etching.

Figure 1 shows schematics of the proposed processes, which comprises two wet-etching steps [26]. The first etching step is an anisotropic silicon etching process that involves KOH (Figure 1a). The details of the microtip mask design and KOH etching theory can be found in [27,28]. A silicon nitride layer is patterned into an array of square shapes with side length *L*, and serves as the etching mask. A pyramidal tip can be generated below each square shape by carefully controlling the etching time. The height (*H*) and the base width (*W*) of the pyramidal microtips depend on the side length (*L*) of the square pattern of the etching mask. Increasing the tip height increases the pitch distance between tips and decreases the tip density per unit area.

The idea for single-sided barbed microtips originated from fish hooks. The sharp tip penetrates into soft material and the barbs increase the resistance to detachment. Figure 1b shows the second etching step for creating microtip barbs. The barb on each tip is formed using isotropic silicon wet etching and HF/HNO_3_. A detailed description of using wet chemical silicon etching in a HF-rich HF/HNO_3_ mixture can be found in [29]. Wet chemical etching of silicon, using HNO_3_-rich HF/HNO_3_ mixtures, was also studied in [30]. SU-8 is employed as the etching mask for fabricating barbs. The etching openings are lithographically patterned in arc or semicircle shapes.

**Figure 2.**
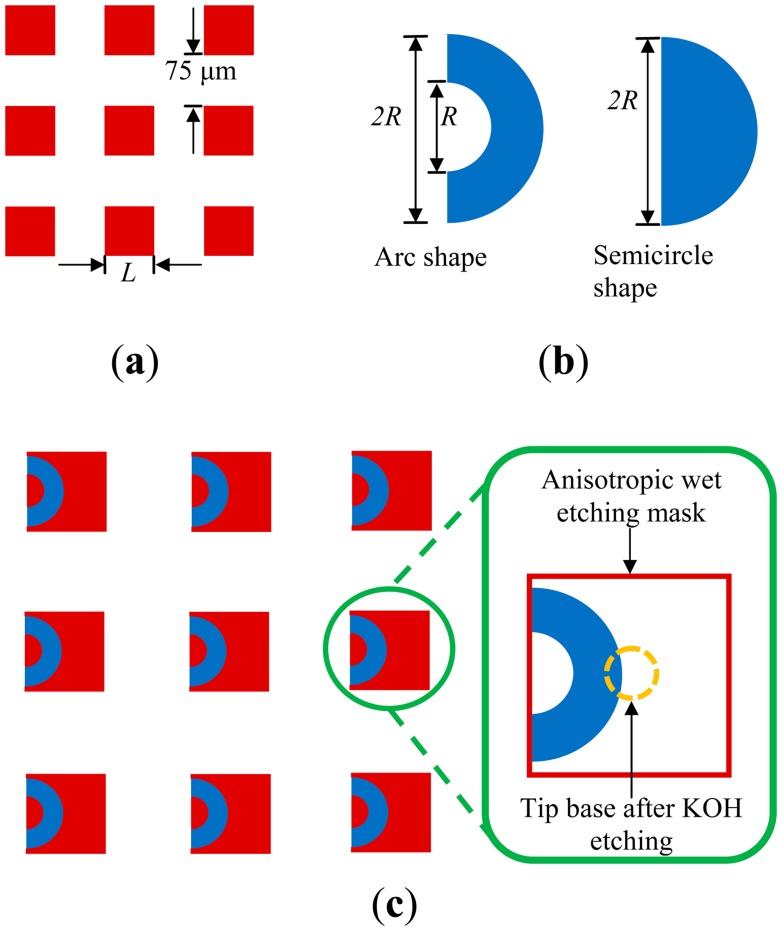
Etching mask design patterns for (**a**) anisotropic wet etching and (**b**) isotropic wet etching; (**c**) The schematic of the combined mask patterns.

Figure 2 shows the etching mask patterns for both the anisotropic and isotropic wet etching processes. Figure 2a shows that an array of solid squares (side length *L*) was used as the anisotropic wet etching mask pattern (silicon nitride). The gap between each square is 75 μm. Figure 2b illustrates the arc and semicircle openings for the etching mask used in isotropic wet etching. The radius of the semicircle and the arc is *R*. In this work, *R* is equal to ((*L*/2) − 20) μm. Figure 2c shows overlapped schematics for the mask patterns of the square and arc arrays. The etching openings (the arc array) are placed near the pyramidal microtip base as indicated in the inset. The etching openings can arranged in various locations, allowing the tip barbs to point in various directions. The side length *L* will affect the maximum height of the tip. Increasing *L* will increase the height of the tip, while decrease the tip density (the number of tips in a unit area). In order to reduce the impedance due to the epidermal layer as well as enhance the attachment of a tip array and human skin, we prefer to have a large tip height. However, the tip height should be less than the thickness of the epidermal layer for avoiding pains when a tip-array attaches human skin. As shown in Table 1, a side length *L* of 300 μm will give a fabricated tip with a height of about 155 μm (less than the thickness of the epidermal layer [31]).

**Figure 3.**
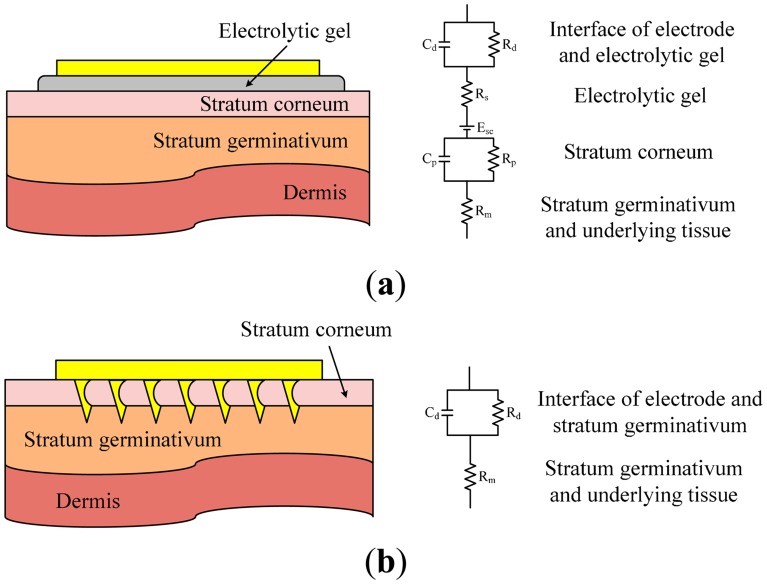
Cross-sectional schematic and simplified electrical model of (**a**) conventional wet electrodes and (**b**) barbed microtip-based dry electrodes.

Figure 3 shows the equivalent circuit models for the configurations of using typical wet electrodes and using microtip-based electrodes. The details of these models can be found in [32]. In general, the human skin can be considered as a plenary structure with a few layers. As shown in Figure 3a, the epidermal layer consists of the stratum corneum (SC) the stratum germinativum (SG). The dermis is below the SG layer. As a traditional wet electrode is attached on the skin, the wet electrode and SC can each be considered parallel RC blocks. The SC layer consists of dead cells and is typically electrically isolative. Therefore, without applying electrolytic gel or abrasion of the SC layer, the impedance between electrode and the skin outer surface could be very high. As the electrolytic gel is applied, the gel will diffuse into the SC so that the conductivity between the skin and the electrode will be enhanced. The electrolytic gel and epidermal layers are considered purely resistive. Because of the electrode-gel interface, a half-cell potential is typically present in the wet electrode configuration.

As shown in Figure 3b, the tips of the dry tip-array electrode penetrate into the SG. SG is an electrically conductive tissue comparable to an electrolyte because SG consists of living cells which is mainly composed of liquid. Since electrical signals bypassing the SC, which can be considered as electrically isolative, the equivalent circuit is relatively simple. Also, the impedance of the configuration with a tip-array electrode is lower compared with the configuration using a traditional wet electrode.

## 3. Electrode Fabrication

**Figure 4.**
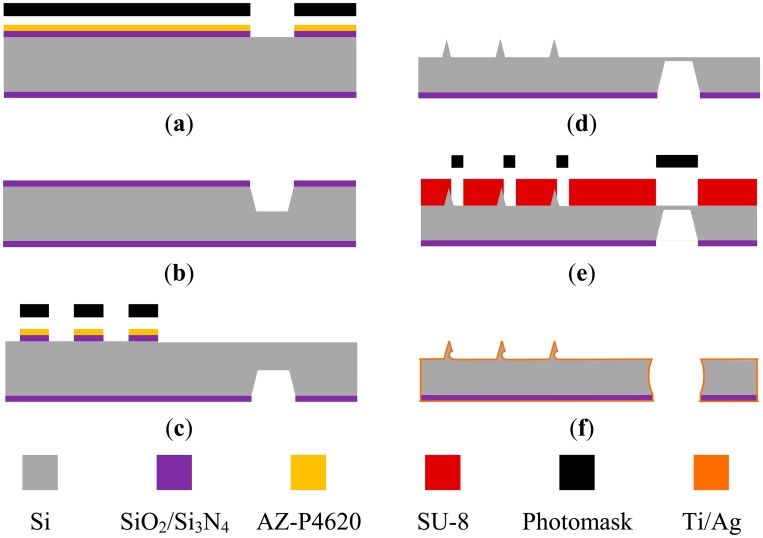
Barbed microtip array fabrication processes.

Figure 4 shows the fabrication process. The starting substrate is a standard, p-type, silicon wafer with (100) orientation. Figure 4a shows the deposition of 4500 Å thick nitride on a 500 Å oxide layer by using low pressure chemical vapor deposition. On the back side, using AZ-P4620 photoresist as the mask, the oxide and nitride layers were patterned as a square opening using RIE. The KOH etchant was mixed with IPA (KOH:DI water:IPA = 130 g:75 mL:4 mL, at 85 °C with constant agitation) and used to fabricate a cavity on the back side (Figure 4b). In subsequent steps, this cavity will be etched to form a through hole that improves the electrical conductivity between the tip array (on the front side) and the electrical lead (on the back side). IPA was employed to reduce the roughness of the etched surface. On the front side, oxide and nitride layers were patterned as square etching masks for generating tips (Figure 4c). The dimensions of these square patterns determine the geometry of each tip and the pitch of the tip array. A preliminary pyramidal microtip array was fabricated using the KOH etchant (Figure 4d). The cavity on the back side is etched during this step; however, a through hole is not yet formed to avoid unnecessary difficulties in the subsequent photolithography step.

An SU-8 photoresist (SU-8 2050, MicroChem, Westborough, MA, USA) was subsequently spun and patterned as the etching holes of barbs (Figure 4e). The etching holes are located on the waist the pyramidal microtips. Note that the thicknesses of the SU-8 etching masks must be larger than the height of the pyramidal microtip to protect the microtips during the etching process. The barbs on the pyramidal microtips were formed using isotropic silicon etching and HF/HNO_3_ (HF:HNO_3_ = 3:20, with constant agitation at room temperature). Concurrently, a through hole was formed. The radius of barbs curvature strongly depends on the etching time; the longer the isotropic etching time is, the smaller is the radius of the barb curvature. The SU-8 etching mask is easily removed using piranha solution (a 3:1 mixture of concentrated sulfuric acid (H_2_SO_4_) and hydrogen peroxide (H_2_O_2_)). Finally, a 15-nm-thick layer of Ti and a 400-nm-thick Ag layer were sputtered on both sides (Figure 4f). Note that the Ti film was used as the adhesion layer.

**Figure 5.**
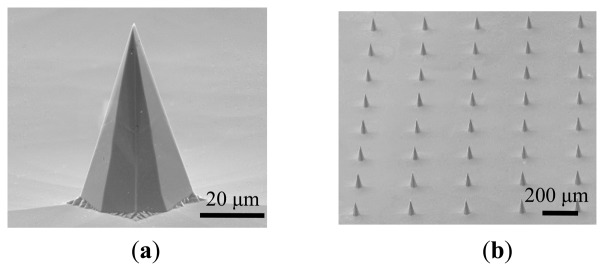
SEM images of the preliminary pyramidal microtips (array) after KOH anisotropic wet etching.

Figure 5 shows SEM images of the fabricated pyramidal microtip (array) after KOH anisotropic wet etching. The tip height is approximately 82 μm and the base width is approximately 42 μm.

**Figure 6.**
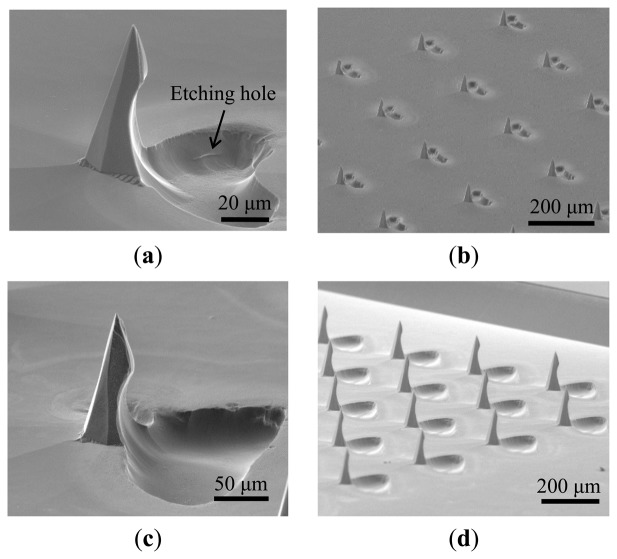
SEM images of the barbed microtips (array) after HF/HNO_3_ isotropic wet etching. (**a**) and (**b**) show the short barbed microtips (82 μm); (**c**) and (**d**) show the long barbed microtips (155 μm).

Figure 6a–d shows SEM images of the 82-μm-high fabricated barbed microtip, the tip array, the 155-μm-high microtip, and the array, respectively. The isotropic etching times for these two types of array were 240 s and 600 s. These figures also indicate uniformity in the fabricated tip arrays. Figure 6a,c shows that isotropic etching generates an etching hole on the substrate. The shape of the etching hole is similar to that of the opening on the etching mask (*i.e.*, the semi-circle or the arc shape shown in Figure 2). Note that barbed microtips were successfully formed with either the arc or semicircle shape openings.

Table 1 lists the etching time (*T*), measured height (*H*), and base width (*W*) of pyramidal tips with various side lengths (*L*) of the square pattern on the etching mask. Figure 1 indicates *L*, *H*, and *W*. The etching times, heights, and base widths of the pyramidal microtips increase as the side length of the square pattern mask increases.

When the side length of the square pattern mask increases to approximately 50 μm, the etching time and tip height typically increase to approximately 120 min and 30 μm, respectively. The aspect ratio of the pyramidal microtip is approximately 1.7.

**Figure 7.**
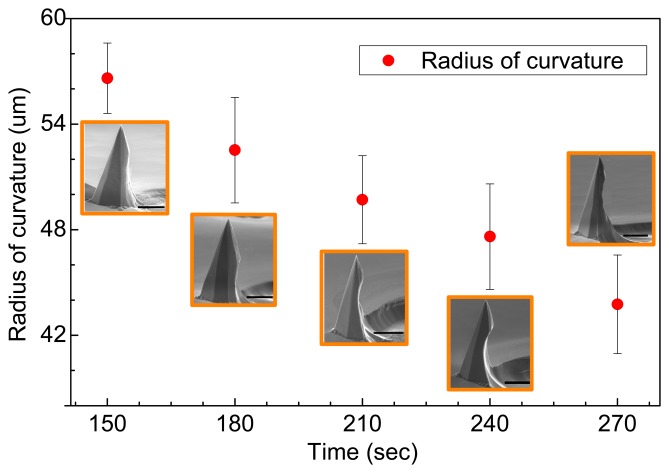
Radius of barb curvature at various etching times (HF/HNO_3_) from 150 to 270 s. The scale bar is 20 μm.

Figure 7 shows the radii of curvature of the barbs, which are estimated by SEM images, for different isotropic etching times. For each etching time (red dot), 10 measurements were performed to obtain a mean value. Figure 7 also shows SEM images of the etched microtips at various etching times. At approximately 150 s of etching, barbs gradually form on the microtips; the optimal barb shape is formed at approximately 240 s. The radius of curvature at the barb decreases almost linearly as the etching time increases. The error bars indicate the standard deviation of the measured values.

**Figure 8.**
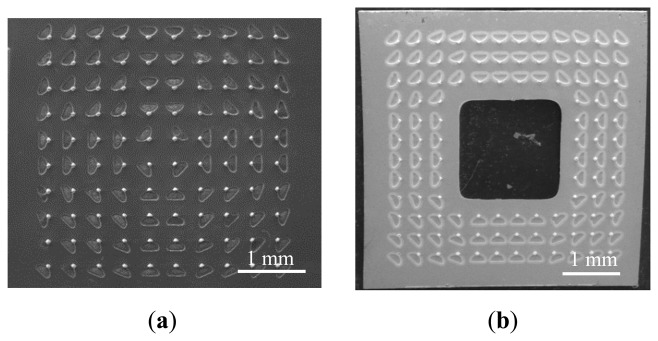

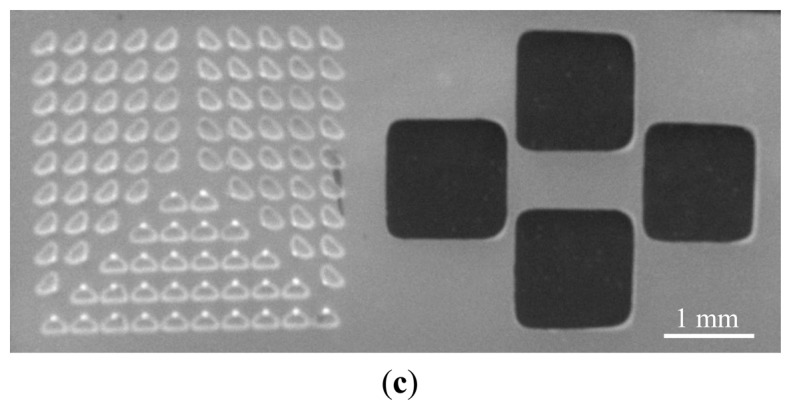
Tip array SEM images: (**a**) without TSV; (**b**) with TSV at the array center; and (**c**) with TSV beside the array. The scale bar is 1 mm.

Figure 8 shows the SEM images of tip arrays with barbs. Figure 8a is the array without TSV. Figure 8b and Figure 8c are the arrays with TSV at different locations.

## 4. Measurement and Discussion

### 4.1. Detaching Force Measurement

**Figure 9.**
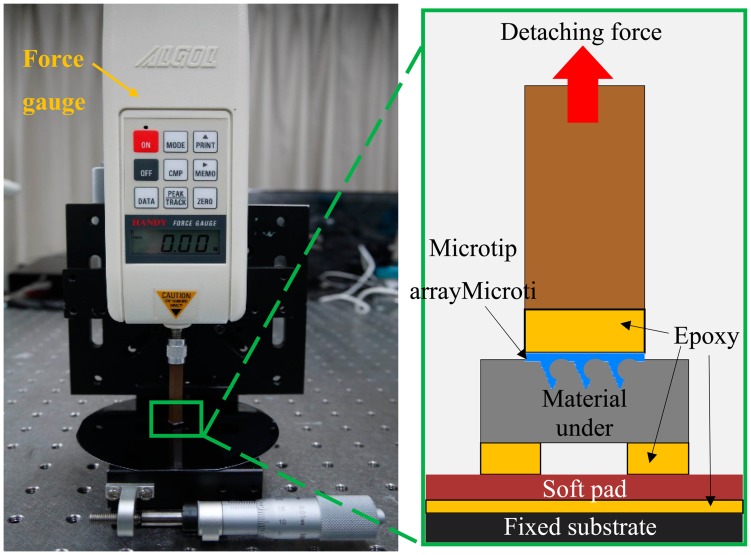
Experimental setup for measuring the force required to detach 10 × 10 microtip arrays from a soft material.

Figure 9 shows the experimental setup used to measure the force required to detach the microtip arrays from various soft materials. A 4 × 4 mm^2^ chip that contained a 10 × 10 array of barbed microtips was glued to the sensing head of the force gauge (HF-1, ALGOL Engineering Co., Taipei, Taiwan). The maximal resolution of the force gauge is 1 mN. The sensing head must be positioned perpendicular to the fixed substrate to avoid measuring unwanted forces in the lateral direction. A soft pad of polydimethylsiloxane (PDMS) was glued between the fixed substrate and the sample material. The force gauge easily records the peak values of the detaching forces as the tip array is pulled up.

**Figure 10.**
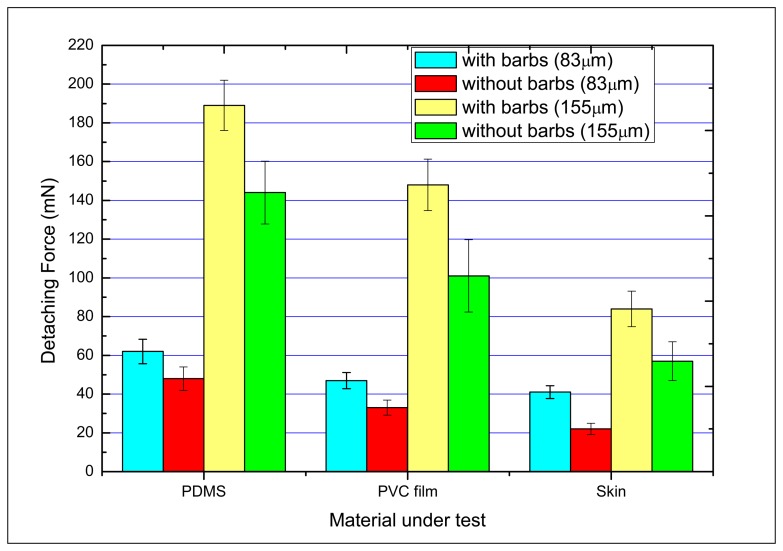
Measured detaching forces of different designs of tips.

The mechanical attachment behaviors of microtip arrays were measured using non-biological materials (PDMS and polyvinylchloride (PVC) film) and human skin. PDMS prepolymer was mixed with a curing agent (Sylgard 184A and 184 B, Dow Corning, Midland, MI, United States) at a 20:1 ratio to obtain a relatively soft PDMS film. The PVC film was a commercially available cling film (Nan-Ya Plastics Corporation, Taipei, Taiwan). The human skin measurement was executed on the back of the hand of a volunteer. Figure 10 presents the detaching force results for 10 × 10 arrays of different designs. Each measured force is the average of 10 measurements. The error bars indicate the standard deviation of the measured values.

The results show that the arrays with larger tip length give larger detaching forces. Also, the detaching forces required for barbed microtip arrays are larger than those for pyramidal microtip arrays. The barbed microtip arrays with tip length of 155 μm detached from the PDMS film yielded the maximal detaching force (189 mN). With the same tip length, the detaching forces for barbed microtip were at least 50% larger than those for non-barbed microtips.

**Figure 11.**
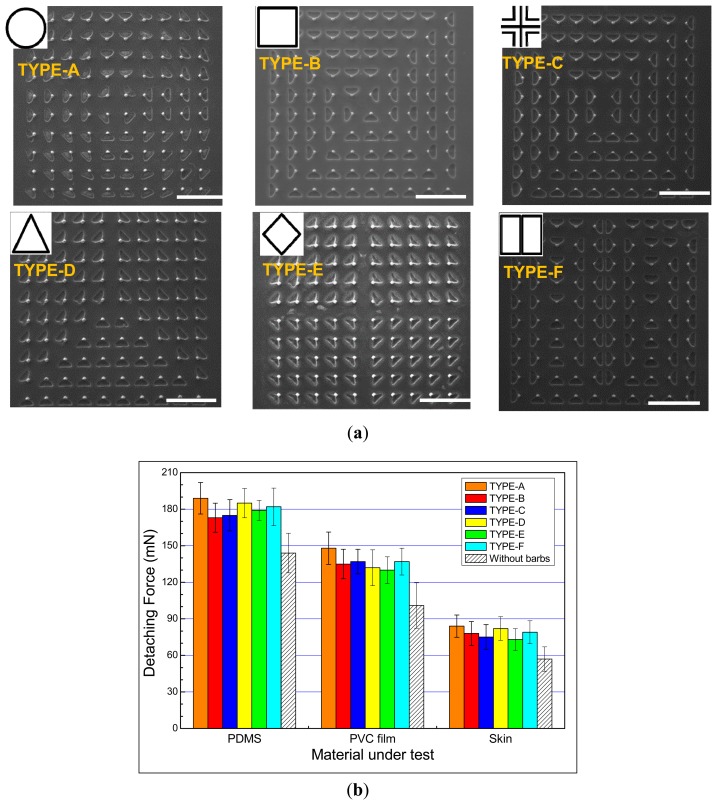
(**a**) SEM images of tip arrays with different array layouts (155 μm tip length). The scale bar is 1 mm; (**b**) Measured detaching forces of the tip arrays with different array layouts.

Six distinct tip array arrangements (*i.e.*, circle, square, cross, triangle, rhombus, rectangle) were designed. Figure 11a shows the top-view SEM images of the arrays with different array layouts. The tip length for these arrays is 155 μm. Figure 11b shows that the detaching force required for the array of the circular array layout was largest compared with the other array layouts. Regardless of array type, the detaching forces required for barbed microtip arrays are always larger than the forces required for non-barbed microtip arrays.

### 4.2. Skin-Electrode Contact Impedance Measurement

**Figure 12.**
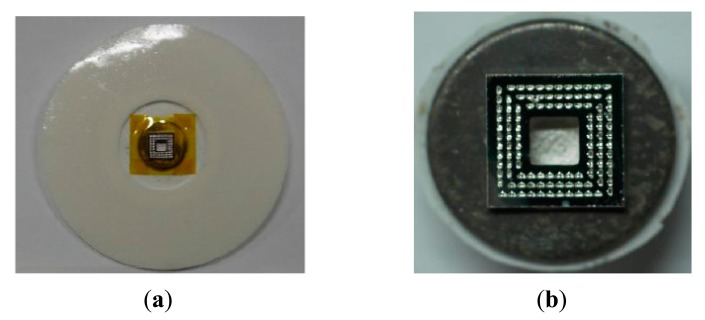
(**a**) Assembled electrode prototype for measuring contact impedance and ECG recording; (**b**) Assembled electrode prototype for EEG recordings.

**Figure 13.**
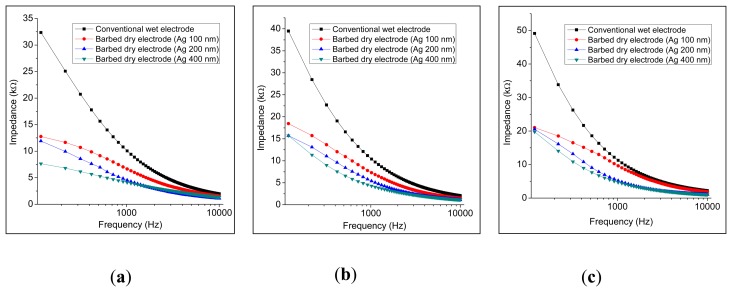
Contact impedances of skin-electrode interface with electrode separation of (**a**) 3 cm; (**b**) 5 cm; and (**c**) 7 cm (center to center).

The impedances of the skin-electrode interface for each electrode type were analyzed using an LCR meter (LCR 6440A, Wayne Kerr Electronics Ltd., London, UK). The type of the assembled electrodes used for measuring the contact impedance is showed in Figure 12a. The tip height and the base width were 155 μm and 86 μm, respectively. During the measurement, two electrodes were placed on the skin at specific separation distances (*i.e.*, center-to-center, 3 cm, 5 cm, and 7 cm) [33,34]. The skin of the participant was cleaned using alcohol. Figure 13a shows the measured impedances *vs.* frequencies for the conventional wet electrode and the barbed dry electrodes at an electrode separation of 3 cm; the microtip-array electrodes clearly yield lower impedance than the wet electrodes do. The microtip-array electrodes deposited with thicker Ag film yielded better (lower) impedances. Similarly, Figure 13b and Figure 13c are the results for the cases with electrode separations of 5 cm and 7 cm.

### 4.3. Electroencephalogram and Electrocardiograph Measurement

To perform biopotential EEG and ECG recordings, the fabricated tip-array electrodes were assembled to a commercially-available wet electrode (Swaromed ECG electrode, Nessler Medizintechnik, Innsbruck, Austria), from which the electrolytic gel was removed. The assembled electrode prototypes for ECG and EEG recordings are shown in Figure 12. Because the metal disk of the wet electrode was larger compared with the tip-array electrode, a trimmed thin polyimide film was used as the insulation layer to avoid allowing the metal disk to contact human skin during measurement.

**Figure 14.**
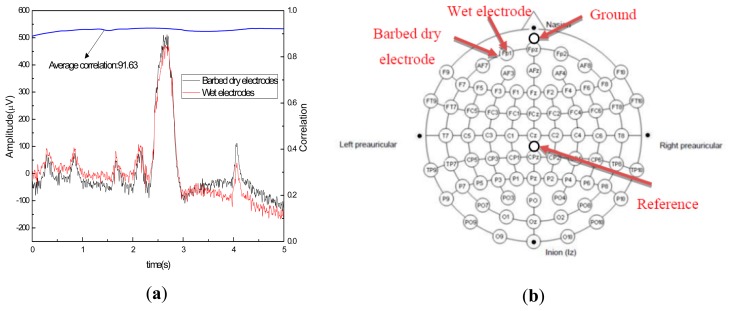
(**a**) EEG signals obtained using the conventional wet electrodes and barbed dry electrodes; (**b**) The locations of the electrodes.

**Figure 15.**
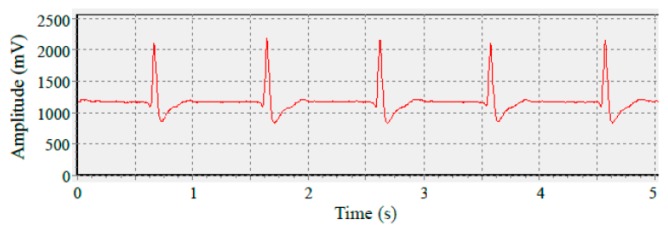

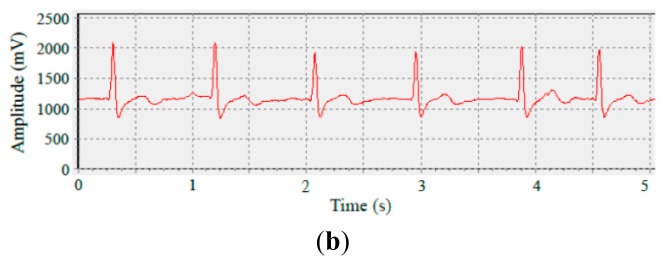
ECG signals obtained using (**a**) the standard wet electrodes; and (**b**) barbed dry electrodes.

For the EEG measurement, the signals were recorded from a young male volunteer by using an EEG amplifier system (NeuroScan, SynAmps RT, 64 channels, Advanced Medical Equipment Ltd., Horsham, UK). The electrodes were attached to the skin near the canthus of the eye by using medical tape. The sampling rate was 500 Hz and the EEG frequency ranges were from 0.1 Hz to 50 Hz. The measurement data were analyzed using EEGLAB [35]. Figure 14a shows the EEG signals measured for the barbed dry microtip-based and conventional wet electrodes. These two curves were simultaneously measured by two channels of the NeuroScan system. The average correlation is about 92%. Figure 14b shows the locations of the electrodes for the EEG recording. The EEG signals were recorded from the forehead (FP1). During the measurement, the participant was asked to blink regularly. The results obtained using the barbed dry electrodes were similar to those obtained using conventional wet electrodes. Regarding ECG testing, a commercially-available ECG recording device was employed. Figure 15a,b shows the ECG results of using wet and barbed dry electrodes, respectively. The figures indicate clear observations of the QRS-complex and T-wave [36,37] cardiac signatures. That is, the barbed dry electrodes record the characteristic ECG peaks relatively effectively.

## 5. Conclusions

This study presented the design, fabrication, and measurement of a barbed microtip-based dry electrode for monitoring biopotential. Fabricating the barbed microtip arrays involved using silicon wet etching techniques. Pyramidal microtip arrays were formed using KOH anisotropic wet etching. The microtips were subsequently reshaped into barbed microtips by using HF/HNO_3_ isotropic wet etching. The etching results yielded by various KOH etching mask designs were analyzed and the radii of the barb curvature were measured. The study involved measuring the forces required to detach the barbed microtip arrays from various materials. The results show that the detaching forces required for barbed microtip arrays are noticeably larger compared with those required for non-barbed arrays and the contact impedance of the barbed dry electrode is lower than is that of the conventional wet electrode that employs skin preparation. Preliminary EEG and ECG recordings of the proposed electrodes yielded adequate signal quality results compared with conventional wet electrodes.

## Figures and Tables

**Table 1.** Pyramidal microtip etching time and the measured KOH etching results for various side lengths (*L*) of the square pattern of the etching mask.

**Mask Pattern Side Length: *L* (μm)**	150	200	250	300	350	400	450	500
**Etching Time: *T* (min)**	85	180	260	390	465	570	690	820
**Pyramidal Tip Height: *H* (μm)**	82 ± 3	98 ± 3	124 ± 3	152 ± 4	180 ± 3	191 ± 3	222 ± 2	252 ± 3
**Pyramidal Tip Base Width: *W* (μm)**	42 ± 2	53 ± 2	72 ± 2	86 ± 2	102 ± 1	117 ± 1	132 ± 2	146 ± 1
